# Engineering Extracellular Matrix Proteins to Enhance Cardiac Regeneration After Myocardial Infarction

**DOI:** 10.3389/fbioe.2020.611936

**Published:** 2021-01-20

**Authors:** Hamid Esmaeili, Chaoyang Li, Xing Fu, Jangwook P. Jung

**Affiliations:** ^1^Department of Biological Engineering, Louisiana State University, Baton Rouge, LA, United States; ^2^School of Animal Sciences, Louisiana State University AgCenter, Baton Rouge, LA, United States

**Keywords:** extracellular matrix, cardiomyocyte proliferation, myocardial infarction, cardiac repair, acellular therapeutics

## Abstract

Engineering microenvironments for accelerated myocardial repair is a challenging goal. Cell therapy has evolved over a few decades to engraft therapeutic cells to replenish lost cardiomyocytes in the left ventricle. However, compelling evidence supports that tailoring specific signals to endogenous cells rather than the direct integration of therapeutic cells could be an attractive strategy for better clinical outcomes. Of many possible routes to instruct endogenous cells, we reviewed recent cases that extracellular matrix (ECM) proteins contribute to enhanced cardiomyocyte proliferation from neonates to adults. In addition, the presence of ECM proteins exerts biophysical regulation in tissue, leading to the control of microenvironments and adaptation for enhanced cardiomyocyte proliferation. Finally, we also summarized recent clinical trials exclusively using ECM proteins, further supporting the notion that engineering ECM proteins would be a critical strategy to enhance myocardial repair without taking any risks or complications of applying therapeutic cardiac cells.

## Introduction

Post-natal cardiomyocytes (CMs) are terminally differentiated cells in the heart and lack proliferative capacity. A withdrawal from the cell-cycle correlates with multinucleated and polyploid CMs (Derks and Bergmann, 2020). At the time of birth, the neonatal human heart comprises primarily mononucleated CMs and ~30% binucleated CMs, and this proportion of mononucleated and binucleated CMs does not change significantly after birth. The DNA of most nuclei is duplicated to become mononucleated tetraploid in childhood when the cells undergo hypertrophy (Bergmann et al., 2009). The overall arrest in CM division is due to the downregulation of cell cycle regulators (Walsh et al., 2010; Zebrowski et al., 2015).

After myocardial infarction (MI), several strategies to replenish lost CMs have been proposed. Engraftment of exogenous cells to restore the damaged myocardium is an attractive remedy to mitigate the progression of cardiac fibrosis. Exogenous sources include stem cell-derived CMs or cardiac progenitor cells. The first-generation cell therapy employed mesenchymal stem cells (MSCs) or similar derivatives (Perin et al., 2015), and the second-generation cell therapy (Cambria et al., 2017) utilized pluripotent stem cell (PSC)-derived CM (Chong et al., 2014; Shiba et al., 2016; Liu et al., 2018), which showed a certain extent of success for cardiac repair with stem cells. However, a major problem associated with cell therapy includes relatively low retention and integration of delivered cells, where only 10–15% are retained regardless of the source (Hou et al., 2005), and only about 1% of injected cells remained after 1 month (Nguyen et al., 2016). In addition, significant arrhythmia, excessive immunosuppression, and potential teratoma formation are the major roadblocks of applying therapeutic cells toward clinical application (Berry et al., 2019; Bolli and Wysoczynski, 2019). Alternatively, cell therapy with endogenous resources is a preferred strategy. However, the contribution of endogenous cardiac stem cells is proven to be minimal, and 31 associated publications from the Anversa laboratory were retracted (Chien et al., 2019), leading to a pause on a clinical trial involving c-kit^+^ cardiac stem cells by the National Institutes of Health [National Heart, Lung, and Blood Institute (NHLBI)] as of October 2018. Interestingly, these results can lead to the longstanding notion that it may be possible to achieve cardiac regeneration without physically presenting cells into the injured heart (French and Holmes, 2019). The consistent mismatch between insignificant cellular engraftment and significant functional improvement has led to our assertion that the functional benefits might well be derived from paracrine actions of the transplanted cells (French and Holmes, 2019), which initiated next-generation therapies with *cell-free* approaches (Cambria et al., 2017). Here, we succinctly summarize extracellular matrix (ECM) proteins directly relevant to or instructing CM proliferation to establish better strategies for enhanced cardiac repair.

## Stimulation Signals for Cardiomyocyte Proliferation

### Extracellular Matrices Associated With Cardiomyocyte Proliferation

From many recent studies (Frangogiannis, 2019), the composition and mechanical properties of the ECM (Chaudhuri et al., 2020) may play a critical role in inducing the regeneration of the myocardium (Yahalom-Ronen et al., 2015). The following studies are recent investigations on ECM proteins and their roles in CM proliferation.

### Fibronectin

Fibronectin is a multidomain, high-molecular-weight glycoprotein, present at low levels in the ECM of the healthy heart. Proteomics analysis of ECM compositions with developmental ages showed that collagen I and III and laminins increase gradually from fetal to adult, while fibronectin decreased with development (Williams et al., 2014). *In vivo*, fibronectin is strongly upregulated in the heart after MI (Konstandin et al., 2013a). Thus, short-term induction of fibronectin following myocardial injury may be tied to a beneficial role in cardiac repair by CM proliferation. The same group proved that fibronectin promotes CM hypertrophy by nuclear factor of activated T cell (NFAT) *in vivo* and *in vitro*, while fibronectin attenuates the activation of physiological growth *in vitro* (Konstandin et al., 2013b). Co-culture of mammalian embryonic cardiac fibroblast (cFB) and CM can promote CM proliferation, and fibronectin secreted by embryonic mouse cFB plays a pro-proliferative role in this process (Ieda et al., 2009). The mechanism of CM proliferation is only partially attributed to fibronectin or collagen type III that promoted CM proliferation by activating heparin-binding EGF-like GFs via β1 integrin signaling (Ieda et al., 2009). In zebrafish heart regeneration, loss-of-function approaches indicated that high expression of fibronectin does not remuscularize the heart (Wang et al., 2013), but fibronectin is necessary for functional regeneration by mobilizing and integrating CMs into the injured region. As such, cFB and fibronectin need additional players to proliferate CMs for cardiac repair.

### Periostin

Periostin is a multimodular protein composed of a signal peptide necessary for secretion, a small cysteine-rich module for the formation of multimers via disulfide bonds, four FAS1 (fasciclin-1) domains interacting with integrins, and a hydrophilic C-terminal region known to interact with other ECM proteins (Kii et al., 2010). Periostin is primarily expressed in the developing heart, but not in healthy adult ventricular myocardium (Snider et al., 2008; Hortells et al., 2020). After acute MI, periostin is re-expressed in the infarct border zones by activated cFBs (Kanisicak et al., 2016). It was reported earlier that periostin can switch differentiated mononucleated CMs into the cell cycle and induce cardiac regeneration with improved myocardial function, as evidenced by an increase in DNA synthesis, aurora B kinase detection, and CM cytokinesis (Kühn et al., 2007). Recently, a report investigated the impact of genetic ablation of periostin in neonatal mice following MI and showed that periostin mediates PI3K/GSK3β/cyclin D1 signaling pathway for myocardial regeneration (Chen et al., 2017). However, this conclusion still remains controversial in adult mice with inducible expression of full-length periostin in that periostin is abundant in the infarcted mouse myocardium in the absence of regeneration (Lorts et al., 2009). Moreover, the periostin-induced cell cycle reentry is mediated in an integrin-dependent manner, which may impact other non-CMs expressing integrins (Kühn et al., 2007).

### Agrin

Agrin is a heparan sulfate proteoglycan. It harbors three laminin-globular (LG) domains within its C-terminal region, and the first two LG domains (LG1 and LG2) are sufficient for binding to α-dystroglycan (αDG). Agrin promotes cell cycle reentry in both neonatal and adult mice (Bassat et al., 2017), and a separate study showed that Hippo/Yap signaling is a key signaling mechanism to mediate endogenous CM dedifferentiation and proliferation (Morikawa et al., 2017). *In vitro* administration of C-terminal agrin from post-natal day 1 (P1) increased CM proliferation (Bassat et al., 2017). The injection of recombinant agrin to the myocardium after MI in juvenile and adult mice also induced CM cell cycle reentry in the healthy myocardium adjacent to the infarcted regions, resulting in reduced scar size and improved cardiac function (Bassat et al., 2017). Although a single administration of agrin promotes cardiac regeneration in adult mice after MI, the degree of CM proliferation observed in this model suggests that additional therapeutic mechanisms are required for functional regeneration of the myocardium (Bassat et al., 2017). In a preclinical porcine model of ischemia reperfusion, local (antegrade) delivery of a single dose of recombinant agrin into the infarcted heart resulted in significant improvement in heart function, infarct size, improved angiogenesis, suppressed inflammatory response, and cell cycle reentry (Baehr et al., 2020). Recent studies reported that binding of agrin to αDG could contribute to enhanced CM proliferation. To expedite such changes, modulating the stiffness of microenvironments could synergistically initiate CM proliferation via dedifferentiation–proliferation–redifferentiation of CMs (Yahalom-Ronen et al., 2015; Wang et al., 2017; Judd et al., 2019).

### Slit-2 and Nephronectin

Slit-2 is a neuronal protein and is the only binding partner of αDG with a single LG domain, while two other homologous Slit-1 and Slit-3 are not yet reported to bind to αDG (Wright et al., 2012). Nephronectin is expressed in CMs throughout the heart and is secreted into the cardiac jelly (Patra et al., 2011). From embryonic cFB-derived ECMs, Slit-2 and nephronectin promote CM cytokinesis both *in vitro* and *in vivo* (Wu et al., 2020), but not cell cycle entry of post-natal CMs. The authors postulated that Slit-2 and nephronectin may act directly on CM and activate intracellular signaling pathways, such as RhoA (Backer et al., 2018).

### Decellularized Extracellular Matrix

Instead of a single ECM component, decellularized zebrafish cardiac ECM (zECM) was intramyocardially injected to treat adult mice after MI (Chen et al., 2016). Given the high regenerative capacity of adult zebrafish hearts, decellularized zECM made from normal or healing hearts can induce mammalian heart regeneration. In a mouse model of acute MI, although a single injection of both normal and healing zECM improved cardiac functional recovery and repair, the healing zECM induced better improvements on heart function. Groups treated with zECM exhibited proliferation of the remaining CMs and multiple cardiac precursor cell populations and reactivation of ErbB2 expression in CMs. NRG1, a mitogen of CMs and a ligand of ErbB2/ErbB4 complex, was detected in zECM but only minimally in murine ECM. The presence of NRG1 in zECM and the reactivation of its receptor ErbB2 in zECM-treated hearts are consistent with the observed proliferation of CMs and improvement of cardiac function. In addition, decellularized porcine myocardial-derived ECM hydrogels were developed (Seif-Naraghi et al., 2013) and showed increases in cardiac muscle and improvements in cardiac function following an injection into the infarct (Christman, 2019). Application of decellularized porcine cardiac ECM to cardiac explant (post-natal day 1) with simultaneous modulation of stiffness using BAPN (3-aminopropionitrile) and ribose (stiffening) (Wang et al., 2020) presented a case that both ECM proteins and mechanical properties of microenvironments are important modulators for cardiac regeneration. Thus, these cases indicate that cardiac ECM-based therapeutics needs to combine with biomechanical modification.

### Microenvironmental Contribution to Cardiomyocyte Proliferation

In addition to CM proliferation via cell cycle reentry, heart regeneration requires dedifferentiation, which indirectly initiates proliferation, and the migration of CM to the injured sites, followed by redifferentiation. Clinically, mechanical unloading of diseased hearts can improve adverse remodeling and improve metabolism (Uriel et al., 2018). The stiffness of the ECM in the myocardium increases progressively, which is correlated with CM cell cycle arrest. By modulating the stiffness of polydimethylsiloxane (PDMS) substrates, compliant (5 kPa) substrates promoted dedifferentiation and proliferation of neonatal CMs including a disorganized sarcomere network and conspicuous cell cycle reentry (Yahalom-Ronen et al., 2015). In contrast, rigid (2 MPa) substrates facilitated karyokinesis (nuclear division) leading to binucleation. Thus, the compliant microenvironment could facilitate CM dedifferentiation and proliferation via its effect on the organization of the cytoskeleton (Yahalom-Ronen et al., 2015). In addition to the first report of neonatal (up to 7 days post-partum) cardiac regeneration (Porrello et al., 2011), another recent investigation found that neonatal regeneration sharply declines within 48 h, with hearts of 2-days-old mice responding to amputation with fibrosis, rather than regeneration (Notari et al., 2018). By comparing the global transcriptomes of mouse hearts at P1 and P2, the authors reported that most differentially expressed transcripts encode ECM proteins and structural constituents of the cytoskeleton. Pharmacological inhibition of the cross-linked enzyme LOX (lysyl oxidase) using BAPN rescued the ability of heart regeneration after apical amputation in P3 neonatal mice. On the other hand, stiffer substrates (10 to 50 kPa) were shown to increase CM proliferation and Yap activity in cultures of β-catenin double-knockout CMs (αE-catenin and αT-catenin), indicating that stabilizing cytoskeleton stimulates the nuclear translocation of Yap (Vite et al., 2018). The differences between published works may be attributed to varying experimental techniques including dimensionality, tissue vs. culture conditions, and stiffness range.

## Clinical Application of Extracellular Matrix-Based Biomaterials for Cardiac Repair

Here are a few examples for ECM-based biomaterials specifically for cardiac repair. More acellular injectable biomaterials for treating MI are reviewed elsewhere (Hernandez and Christman, 2017; Christman, 2019). In addition, commercially available ECM-based scaffolds for cardiac repair are discussed in the recent reviews therein (Swinehart and Badylak, 2016; Pattar et al., 2019)

CorMatrix is a scaffold derived from small intestinal submucosa (SIS) and is the most widely used SIS-ECM product in cardiovascular surgery, which also recently received Food and Drug Administration (FDA) approval (Mosala Nezhad et al., 2016). CorMatrix ECM cardiac patches were tested in clinical trials (ClinicalTrials.gov identifier: NCT02887768), claiming to promote endogenous cardiac regeneration. However, a study utilizing CorMatrix patches in infants with congenital heart disease did not show evidence of native cardiac tissue ingrowth within 21 months (Nelson et al., 2016). Further complications were reported from other clinical trials with a CorMatrix patch, including patch dehiscence after atrioventricular continuity reconstruction following massive posterior annulus decalcification and mitral valve replacement for mitral stenosis due to dystrophic calcification (Poulin et al., 2013). These results suggest that CorMatrix may elicit eosinophilic inflammation in human patients after implantation, perhaps via α-gal (galactose-α-1,3-galactose) present in the porcine intestine (Mosala Nezhad et al., 2016), which probably supports the notion that completely defined therapeutics would be beneficial to avoid adverse reactions in human patients.

VentriGel is an ECM hydrogel derived from decellularized porcine myocardium (Singelyn et al., 2012; Seif-Naraghi et al., 2013; Hernandez and Christman, 2017) examined in a recently published clinical trial (ClinicalTrials.gov identifier: NCT02305602). The outcomes of the first-in-man trial highlighted the safety and efficacy of the treatment over 6 months (Traverse et al., 2019). VentriGel is a relatively weak hydrogel (Johnson et al., 2011), exhibiting two orders of magnitude lower stiffness than the stiffness (13 Pa of storage modulus at 8 mg/mL) of healthy, normal adult myocardium (around 10–15 kPa; Pandey et al., 2018). While there is yet sufficient evidence for the capability of decellularized hydrogel to promote endogenous cardiac regeneration, the ECM signals of normal healthy myocardium can prove a promising strategy for engineering biomaterials for cardiac repair.

## Conclusions and Outlook

From earlier studies treating p38 mitogen-activated protein (MAP) kinase inhibitor (SB203580) for CM mitosis (Engel et al., 2005, 2006), a number of stimulation signals have been identified for CM to reenter cell cycle and to promote [cyclin A2 (Shapiro et al., 2014); a cocktail of CDK1, CDK4, cyclin D1, and B1 (Mohamed et al., 2018); Tbx20 (Xiang et al., 2016); and hypoxia-inducible factor 1α (HIF1α) (Guimarães-Camboa et al., 2015)] or inhibit [Meis1 (Mahmoud et al., 2013) and thyroid hormone (Hirose et al., 2019)] preexisting CM proliferation. Hippo (Heallen et al., 2011; Leach et al., 2017) and NRG1/ErbB4 (D'uva et al., 2015) pathways could be a molecular strategy to promote adult CM proliferation. The Hippo-DGC (dystrophin–glycoprotein complex)-agrin studies identified that viral delivery or a direct injection of CM proliferation agonist could be a viable cardiac repair strategy (Morikawa et al., 2017). More recently, ERBB2-ERK (extracellular signal-regulated kinase)-YAP mechanotransduction signaling was shown to trigger CM mitosis and epithelial-to-mesenchymal (EMT)-like transition toward phenotypic plasticity (Aharonov et al., 2020). Another important consideration is to exploit the metabolic switch from mitochondrial oxidative phosphorylation to glycolysis to induce CM proliferation. Recent studies reported that CM proliferation can be enhanced by inhibiting fatty-acid utilization with deletion of pyruvate dehydrogenase kinase-4 (PDK4) (Cardoso et al., 2020), activating Nrg1/ErbB2 signaling (Honkoop et al., 2019), and activating PPARδ/PDK1/p308Akt/GSK3β/β-catenin-pathway (Magadum et al., 2017).

Translation of technologies to augment the stimulation signals requires a thoughtful examination, especially considering the oncogenic potential of activating growth pathways (Heallen et al., 2019). Another promising strategy of activating CM proliferation is to deliver an intrinsic extracellular factor (e.g., FSTL-1) via an engineered patch to stimulate endogenous repair (Wei et al., 2015). This acellular approach reduces the laborious effort to prepare therapeutic cells, while avoiding potential tumor formation and adverse immune rejection from the patient. However, such an extracellular factor can also potentially stimulate non-CMs. Specific ligands that only allow engagement with CMs are needed to avoid adverse activation of the expansion of cFBs and their differentiation into myofibroblasts (specifically associated with fibrosis) (Fu et al., 2018).

**Figure 1:**
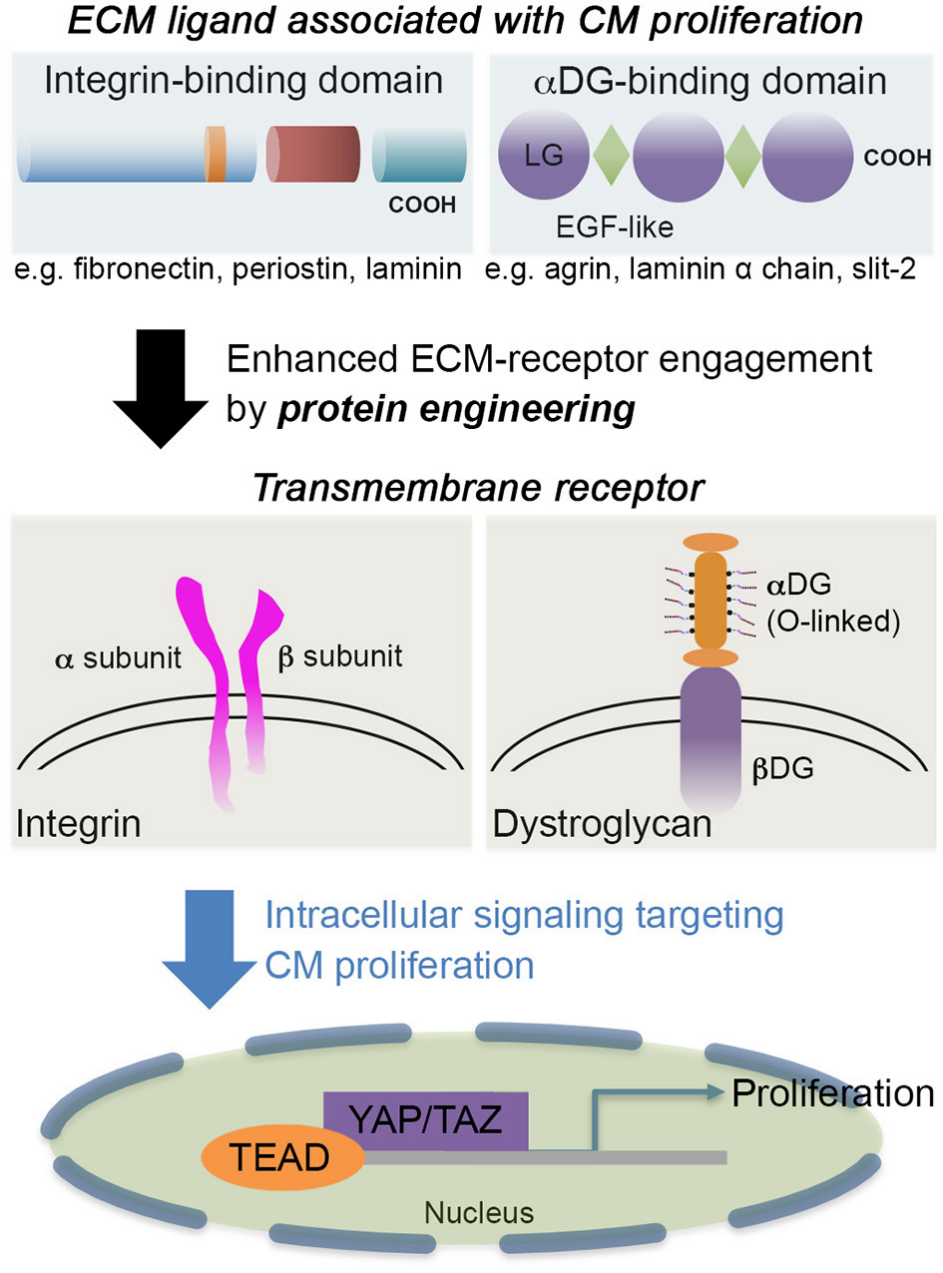
The schematic showcases an example of integrin-binding domain, such as fibronectin, periostin, or laminin; and αDG-binding domain, such as agrin, Slit-2, or laminin α chains. The engagement of extracellular matrix (ECM) proteins via transmembrane receptors [integrin or dystroglycan (DG)] can be tuned by *engineering* the ECM proteins to further stimulate cardiomyocyte (CM) proliferation and associated pathways without penetrating CMs. Some laminin-globular (LG) domains from laminins are also interactive with integrin (Aumailley, 2013) as well as heparin (Ishihara et al., 2018), so the receptor-ligand integrations are not exclusive. For CM proliferation, active (unphosphorylated) Yap is translocated to the nucleus where the Yap interacts with TEA domain (TEAD) transcription factor to regulate cell proliferation.

Laminin α chains and several proteoglycans harbor a few tandem arrays of LG domains. Despite the structural similarity between agrin and laminin, binding affinity to αDG and the configuration of a tandem array of LG domains are distinct (Dempsey et al., 2019). This different feature may have conferred the different roles of ECM proteins containing LG domains in CM proliferation and differentiation. Thus, the therapeutic application of LG domain containing ECMs (agrin, laminin, and Slit-2, as depicted in Figure 1) needs to have further specification in their molecular nature and the receptors exclusively expressed in CM (for recent reviews on LG domain containing molecules, see Hohenester, 2019a,b; Yap et al., 2019).

ECM proteins that were applied for myocardial regeneration augmented the additional modification to enhance longevity and contribution for remuscularization (e.g., CorMatrix and VentriGel). ECM proteins binding to αDG, agrin, or Slit-2 contributed to CM proliferation (Bigotti et al., 2020), but further mechanistic understanding requires establishing a better strategy for CM proliferation. In addition to providing novel engineering strategies for cardiac repair, more critical analysis of CM proliferation assays and induction of CM proliferation by microRNA, metabolic switch, or small molecule is necessary to inform the field to efficiently reach the goal of myocardial regeneration (Leone and Engel, 2019). A recent phase I clinical trial (ESCORT, NCT02057900) with fibrin and Matrigel composites incorporating human embryonic stem cell (hESC)-derived cardiac progenitor cells (Isl1^+^) proved the safety of the approach (Menasche et al., 2018; Menasché, 2020), which showed a synergistic contribution to the regeneration of the myocardium without apparent integration of the delivered cardiac cells. Thus, a protein engineering strategy could be a starting point to target a specific receptor and well-defined signal pathways (e.g., Hippo/Yap) (Bassat et al., 2017; Morikawa et al., 2017) (Figure 1). Then, engineering multifactorial, acellular biomaterials with a simple deployment strategy could be a therapeutic goal (Christman, 2019). Ideally, both mechanical compensation and biochemical definition would be necessary. In addition, what levels of complexity we have to address are an important question to answer (Ogle et al., 2016) since a small increase in the ejection fraction of 5–10% in the function of the left ventricle would be a meaningful resolution to mitigate heart failure for patients suffering from post-injury.

## Author Contributions

JJ and XF conceived the overall topics of discussion. All authors wrote, read, and approved the final manuscript.

## Conflict of Interest

The authors declare that the research was conducted in the absence of any commercial or financial relationships that could be construed as a potential conflict of interest.

## References

[B1] Aharonov A. Shakked A. Umansky K. B. Savidor A. Genzelinakh A. Kain D.. (2020). Erbb2 drives yap activation and emt-like processes during cardiac regeneration. Nat. Cell Biol. 22, 1346–1356. 10.1038/s41556-020-00588-4 33046882

[B2] Aumailley M. (2013). The laminin family. Cell Adh. Migr. 7, 48–55. 10.4161/cam.22826 23263632 PMC3544786

[B3] Backer S. Lokmane L. Landragin C. Deck M. Garel S. Bloch-Gallego E. (2018). Trio gef mediates rhoa activation downstream of slit2 and coordinates telencephalic wiring. Development 145:dev153692. 10.1242/dev.153692 30177526

[B4] Baehr A. Umansky K. B. Bassat E. Jurisch V. Klett K. Bozoglu T.. (2020). Agrin promotes coordinated therapeutic processes leading to improved cardiac repair in pigs. Circulation 142, 868–881. 10.1161/CIRCULATIONAHA.119.045116 32508131

[B5] Bassat E. Mutlak Y. E. Genzelinakh A. Shadrin I. Y. Baruch Umansky K. Yifa O.. (2017). The extracellular matrix protein agrin promotes heart regeneration in mice. Nature 547, 179–184. 10.1038/nature22978 28581497 PMC5769930

[B6] Bergmann O. Bhardwaj R. D. Bernard S. Zdunek S. Barnabé-Heider F. Walsh S.. (2009). Evidence for cardiomyocyte renewal in humans. Science 324, 98–102. 10.1126/science.1164680 19342590 PMC2991140

[B7] Berry J. L. Zhu W. Tang Y. L. Krishnamurthy P. Ge Y. Cooke J. P.. (2019). Convergences of life sciences and engineering in understanding and treating heart failure. Circ. Res. 124, 161–169. 10.1161/CIRCRESAHA.118.314216 30605412 PMC6350935

[B8] Bigotti M. G. Skeffington K. L. Jones F. P. Caputo M. Brancaccio A. (2020). Agrin-mediated cardiac regeneration: some open questions. Front. Bioeng. Biotech. 8:594 10.3389/fbioe.2020.00594 PMC7308530 32612983

[B9] Bolli R. Wysoczynski M. (2019). Human embryonic stem cell-derived cardiomyocytes. Circ. Res. 124, 1157–1159. 10.1161/CIRCRESAHA.119.314869 30973816

[B10] Cambria E. Pasqualini F. S. Wolint P. Günter J. Steiger J. Bopp A.. (2017). Translational cardiac stem cell therapy: advancing from first-generation to next-generation cell types. NPJ Regen. Med. 2:17. 10.1038/s41536-017-0024-1 29302353 PMC5677990

[B11] Cardoso A. C. Lam N. T. Savla J. J. Nakada Y. Pereira A. H. M. Elnwasany A.. (2020). Mitochondrial substrate utilization regulates cardiomyocyte cell cycle progression. Nat. Metab. 2, 167–178. 10.1038/s42255-020-0169-x 32617517 PMC7331943

[B12] Chaudhuri O. Cooper-White J. Janmey P. A. Mooney D. J. Shenoy V. B. (2020). Effects of extracellular matrix viscoelasticity on cellular behaviour. Nature 584, 535–546. 10.1038/s41586-020-2612-2 32848221 PMC7676152

[B13] Chen W. C. Wang Z. Missinato M. A. Park D. W. Long D. W. Liu H. J.. (2016). Decellularized zebrafish cardiac extracellular matrix induces mammalian heart regeneration. Sci. Adv. 2:e1600844. 10.1126/sciadv.1600844 28138518 PMC5262469

[B14] Chen Z. Xie J. Hao H. Lin H. Wang L. Zhang Y.. (2017). Ablation of periostin inhibits post-infarction myocardial regeneration in neonatal mice mediated by the phosphatidylinositol 3 kinase/glycogen synthase kinase 3β/cyclin d1 signalling pathway. Cardiovasc. Res. 113, 620–632. 10.1093/cvr/cvx001 28453729 PMC5412017

[B15] Chien K. R. Frisen J. Fritsche-Danielson R. Melton D. A. Murry C. E. Weissman I. L. (2019). Regenerating the field of cardiovascular cell therapy. Nat. Biotech. 37, 232–237. 10.1038/s41587-019-0042-1 30778231

[B16] Chong J. J. H. Yang X. Don C. W. Minami E. Liu Y.-W. Weyers J. J. (2014). Human embryonic-stem-cell-derived cardiomyocytes regenerate non-human primate hearts. Nature 510, 273–277. 10.1038/nature13233 24776797 PMC4154594

[B17] Christman K. L. (2019). Biomaterials for tissue repair. Science 363, 340–341. 10.1126/science.aar2955 30679357 PMC6697375

[B18] Dempsey C. E. Bigotti M. G. Adams J. C. Brancaccio A. (2019). Analysis of alpha-dystroglycan/lg domain binding modes: investigating protein motifs that regulate the affinity of isolated lg domains. Front. Mol. Biosci. 6:18. 10.3389/fmolb.2019.00018 30984766 PMC6450144

[B19] Derks W. Bergmann O. (2020). Polyploidy in cardiomyocytes: roadblock to heart regeneration? Circ. Res. 126, 552–565. 10.1161/CIRCRESAHA.119.315408 32078450

[B20] D'uva G. Aharonov A. Lauriola M. Kain D. Yahalom-Ronen Y. Carvalho S.. (2015). Erbb2 triggers mammalian heart regeneration by promoting cardiomyocyte dedifferentiation and proliferation. Nat. Cell Biol. 17, 627–638. 10.1038/ncb3149 25848746

[B21] Engel F. B. Hsieh P. C. Lee R. T. Keating M. T. (2006). Fgf1/p38 map kinase inhibitor therapy induces cardiomyocyte mitosis, reduces scarring, and rescues function after myocardial infarction. Proc. Natl. Acad. Sci. U.S.A. 103, 15546–15551. 10.1073/pnas.0607382103 17032753 PMC1622860

[B22] Engel F. B. Schebesta M. Duong M. T. Lu G. Ren S. Madwed J. B.. (2005). P38 map kinase inhibition enables proliferation of adult mammalian cardiomyocytes. Genes Dev. 19, 1175–1187. 10.1101/gad.1306705 15870258 PMC1132004

[B23] Frangogiannis N. G. (2019). The extracellular matrix in ischemic and nonischemic heart failure. Circ. Res. 125, 117–146. 10.1161/CIRCRESAHA.119.311148 31219741 PMC6588179

[B24] French B. A. Holmes J. W. (2019). Implications of scar structure and mechanics for post-infarction cardiac repair and regeneration. Exp. Cell Res. 376, 98–103. 10.1016/j.yexcr.2019.01.001 30610848

[B25] Fu X. Khalil H. Kanisicak O. Boyer J. G. Vagnozzi R. J. Maliken B. D.. (2018). Specialized fibroblast differentiated states underlie scar formation in the infarcted mouse heart. J. Clin. Invest. 128, 2127–2143. 10.1172/JCI98215 29664017 PMC5957472

[B26] Guimarães-Camboa N. Stowe J. Aneas I. Sakabe N. Cattaneo P. Henderson L.. (2015). Hif1α represses cell stress pathways to allow proliferation of hypoxic fetal cardiomyocytes. Dev. Cell 33, 507–521. 10.1016/j.devcel.2015.04.021 26028220 PMC4509618

[B27] Heallen T. Zhang M. Wang J. Bonilla-Claudio M. Klysik E. Johnson R. L.. (2011). Hippo pathway inhibits wnt signaling to restrain cardiomyocyte proliferation and heart size. Science 332, 458–461. 10.1126/science.1199010 21512031 PMC3133743

[B28] Heallen T. R. Kadow Z. A. Kim J. H. Wang J. Martin J. F. (2019). Stimulating cardiogenesis as a treatment for heart failure. Circ. Res. 124, 1647–1657. 10.1161/CIRCRESAHA.118.313573 31120819 PMC6534162

[B29] Hernandez M. J. Christman K. L. (2017). Designing acellular injectable biomaterial therapeutics for treating myocardial infarction and peripheral artery disease. JACC Basic Transl. Sci. 2, 212–226. 10.1016/j.jacbts.2016.11.008 29057375 PMC5646282

[B30] Hirose K. Payumo A. Y. Cutie S. Hoang A. Zhang H. Guyot R.. (2019). Evidence for hormonal control of heart regenerative capacity during endothermy acquisition. Science 364, 184–188. 10.1126/science.aar2038 30846611 PMC6541389

[B31] Hohenester E. (2019a). Laminin g-like domains: dystroglycan-specific lectins. Curr. Opin. Struct. Biol. 56, 56–63. 10.1016/j.sbi.2018.11.007 30530204 PMC6925595

[B32] Hohenester E. (2019b). Structural biology of laminins. Essays Biochem. 63, 285–295. 10.1042/EBC20180075 31092689 PMC6744579

[B33] Honkoop H. De Bakker D. E. Aharonov A. Kruse F. Shakked A. Nguyen P. D.. (2019). Single-cell analysis uncovers that metabolic reprogramming by erbb2 signaling is essential for cardiomyocyte proliferation in the regenerating heart. Elife 8:e50163. 10.7554/eLife.50163.sa2 31868166 PMC7000220

[B34] Hortells L. Valiente-Alandi I. Thomas Z. M. Agnew E. J. Schnell D. J. York A. J.. (2020). A specialized population of periostin-expressing cardiac fibroblasts contributes to postnatal cardiomyocyte maturation and innervation. Proc. Natl. Acad. Sci. U.S.A. 117, 21469–21479. 10.1073/pnas.2009119117 32817558 PMC7474678

[B35] Hou D. Youssef E. A. Brinton T. J. Zhang P. Rogers P. Price E. T.. (2005). Radiolabeled cell distribution after intramyocardial, intracoronary, and interstitial retrograde coronary venous delivery: implications for current clinical trials. Circulation 112, I150–I156. 10.1161/CIRCULATIONAHA.104.526749 16159808

[B36] Ieda M. Tsuchihashi T. Ivey K. N. Ross R. S. Hong T. T. Shaw R. M.. (2009). Cardiac fibroblasts regulate myocardial proliferation through beta1 integrin signaling. Dev. Cell 16, 233–244. 10.1016/j.devcel.2008.12.007 19217425 PMC2664087

[B37] Ishihara J. Ishihara A. Fukunaga K. Sasaki K. White M. J. V. Briquez P. S.. (2018). Laminin heparin-binding peptides bind to several growth factors and enhance diabetic wound healing. Nat. Commun. 9:2163. 10.1038/s41467-018-04525-w 29867149 PMC5986797

[B38] Johnson T. D. Lin S. Y. Christman K. L. (2011). Tailoring material properties of a nanofibrous extracellular matrix derived hydrogel. Nanotechnology 22:494015. 10.1088/0957-4484/22/49/494015 22101810 PMC3280097

[B39] Judd J. Lovas J. Huang G. N. (2019). Defined factors to reactivate cell cycle activity in adult mouse cardiomyocytes. Sci. Rep. 9:18830. 10.1038/s41598-019-55027-8 31827131 PMC6906479

[B40] Kanisicak O. Khalil H. Ivey M. J. Karch J. Maliken B. D. Correll R. N.. (2016). Genetic lineage tracing defines myofibroblast origin and function in the injured heart. Nat. Commun. 7:12260. 10.1038/ncomms12260 27447449 PMC5512625

[B41] Kii I. Nishiyama T. Li M. Matsumoto K. Saito M. Amizuka N.. (2010). Incorporation of tenascin-c into the extracellular matrix by periostin underlies an extracellular meshwork architecture. J. Biol. Chem. 285, 2028–2039. 10.1074/jbc.M109.051961 19887451 PMC2804360

[B42] Konstandin M. H. Toko H. Gastelum G. M. Quijada P. De La Torre A. Quintana M.. (2013a). Fibronectin is essential for reparative cardiac progenitor cell response after myocardial infarction. Circ. Res. 113, 115–125. 10.1161/CIRCRESAHA.113.301152 23652800 PMC3815660

[B43] Konstandin M. H. Völkers M. Collins B. Quijada P. Quintana M. De La Torre A.. (2013b). Fibronectin contributes to pathological cardiac hypertrophy but not physiological growth. Basic Res. Cardiol. 108:375. 10.1007/s00395-013-0375-8 23912225 PMC3813434

[B44] Kühn B. Del Monte F. Hajjar R. J. Chang Y. S. Lebeche D. Arab S.. (2007). Periostin induces proliferation of differentiated cardiomyocytes and promotes cardiac repair. Nat. Med. 13, 962–969. 10.1038/nm1619 17632525

[B45] Leach J. P. Heallen T. Zhang M. Rahmani M. Morikawa Y. Hill M. C.. (2017). Hippo pathway deficiency reverses systolic heart failure after infarction. Nature 550, 260–264. 10.1038/nature24045 28976966 PMC5729743

[B46] Leone M. Engel F. B. (2019). Advances in heart regeneration based on cardiomyocyte proliferation and regenerative potential of binucleated cardiomyocytes and polyploidization. Clin. Sci. 133, 1229–1253. 10.1042/CS20180560 31175264

[B47] Liu Y. W. Chen B. Yang X. Fugate J. A. Kalucki F. A. Futakuchi-Tsuchida A. (2018). Human embryonic stem cell-derived cardiomyocytes restore function in infarcted hearts of non-human primates. Nat. Biotech. 36, 597–605. 10.1038/nbt.4162 PMC6329375 29969440

[B48] Lorts A. Schwanekamp J. A. Elrod J. W. Sargent M. A. Molkentin J. D. (2009). Genetic manipulation of periostin expression in the heart does not affect myocyte content, cell cycle activity, or cardiac repair. Circ. Res. 104, e1–e7. 10.1161/CIRCRESAHA.108.188649 19038863 PMC2644415

[B49] Magadum A. Ding Y. He L. Kim T. Vasudevarao M. D. Long Q.. (2017). Live cell screening platform identifies pparδ as a regulator of cardiomyocyte proliferation and cardiac repair. Cell Res. 27, 1002–1019. 10.1038/cr.2017.84 28621328 PMC5539351

[B50] Mahmoud A. I. Kocabas F. Muralidhar S. A. Kimura W. Koura A. S. Thet S.. (2013). Meis1 regulates postnatal cardiomyocyte cell cycle arrest. Nature 497, 249–253. 10.1038/nature12054 23594737 PMC4159712

[B51] Menasché P. (2020). Cell therapy with human esc-derived cardiac cells: clinical perspectives. Front. Bioeng. Biotechnol. 8:601560. 10.3389/fbioe.2020.601560 33195177 PMC7649799

[B52] Menasche P. Vanneaux V. Hagege A. Bel A. Cholley B. Parouchev A.. (2018). Transplantation of human embryonic stem cell-derived cardiovascular progenitors for severe ischemic left ventricular dysfunction. J. Am. Coll. Cardiol. 71, 429–438. 10.1016/j.jacc.2017.11.047 29389360

[B53] Mohamed T. M. A. Ang Y. S. Radzinsky E. Zhou P. Huang Y. Elfenbein A.. (2018). Regulation of cell cycle to stimulate adult cardiomyocyte proliferation and cardiac regeneration. Cell 173, 104–116.e112. 10.1016/j.cell.2018.02.014 29502971 PMC5973786

[B54] Morikawa Y. Heallen T. Leach J. Xiao Y. Martin J. F. (2017). Dystrophin-glycoprotein complex sequesters yap to inhibit cardiomyocyte proliferation. Nature 547, 227–231. 10.1038/nature22979 28581498 PMC5528853

[B55] Mosala Nezhad Z. Poncelet A. De Kerchove L. Gianello P. Fervaille C. El Khoury G. (2016). Small intestinal submucosa extracellular matrix (cormatrix®) in cardiovascular surgery: a systematic review. Interact. Cardiovasc. Thorac. Surg. 22, 839–850. 10.1093/icvts/ivw020 26912574 PMC4986773

[B56] Nelson J. S. Heider A. Si M. S. Ohye R. G. (2016). Evaluation of explanted cormatrix intracardiac patches in children with congenital heart disease. Ann. Thorac. Surg. 102, 1329–1335. 10.1016/j.athoracsur.2016.03.086 27237540

[B57] Nguyen P. K. Neofytou E. Rhee J. W. Wu J. C. (2016). Potential strategies to address the major clinical barriers facing stem cell regenerative therapy for cardiovascular disease: a review. JAMA Cardiol. 1, 953–962. 10.1001/jamacardio.2016.2750 27579998 PMC5378463

[B58] Notari M. Ventura-Rubio A. Bedford-Guaus S. J. Jorba I. Mulero L. Navajas D.. (2018). The local microenvironment limits the regenerative potential of the mouse neonatal heart. Sci. Adv. 4:eaao5553. 10.1126/sciadv.aao5553 29732402 PMC5931766

[B59] Ogle B. M. Bursac N. Domian I. Huang N. F. Menasché P. Murry C. E.. (2016). Distilling complexity to advance cardiac tissue engineering. Sci. Transl. Med. 8:342ps313. 10.1126/scitranslmed.aad2304 27280684 PMC4959426

[B60] Pandey P. Hawkes W. Hu J. Megone W. V. Gautrot J. Anilkumar N.. (2018). Cardiomyocytes sense matrix rigidity through a combination of muscle and non-muscle myosin contractions. Dev. Cell 45:661. 10.1016/j.devcel.2017.12.024 29870723 PMC5988560

[B61] Patra C. Diehl F. Ferrazzi F. Van Amerongen M. J. Novoyatleva T. Schaefer L.. (2011). Nephronectin regulates atrioventricular canal differentiation via bmp4-has2 signaling in zebrafish. Development 138, 4499–4509. 10.1242/dev.067454 21937601 PMC3253110

[B62] Pattar S. S. Fatehi Hassanabad A. Fedak P. W. M. (2019). Acellular extracellular matrix bioscaffolds for cardiac repair and regeneration. Front. Cell Dev. Biol. 7:63. 10.3389/fcell.2019.00063 31080800 PMC6497812

[B63] Perin E. C. Borow K. M. Silva G. V. Demaria A. N. Marroquin O. C. Huang P. P. (2015). A phase II dose-escalation study of allogeneic mesenchymal precursor cells in patients with ischemic or nonischemic heart failure. Circ. Res. 117, 576–584. 10.1161/CIRCRESAHA.115.306332 26148930

[B64] Porrello E. R. Mahmoud A. I. Simpson E. Hill J. A. Richardson J. A. Olson E. N.. (2011). Transient regenerative potential of the neonatal mouse heart. Science 331, 1078–1080. 10.1126/science.1200708 21350179 PMC3099478

[B65] Poulin F. Horlick E. M. David T. Woo A. Thavendiranathan P. (2013). 3-dimensional transesophageal echocardiography-guided closure of a gerbode shunt due to cormatrix patch dehiscence. J. Am. Coll. Cardiol. 62:e5. 10.1016/j.jacc.2013.02.090 23680256

[B66] Seif-Naraghi S. B. Singelyn J. M. Salvatore M. A. Osborn K. G. Wang J. J. Sampat U.. (2013). Safety and efficacy of an injectable extracellular matrix hydrogel for treating myocardial infarction. Sci. Transl. Med. 5:173ra125. 10.1126/scitranslmed.3005503 23427245 PMC3848875

[B67] Shapiro S. D. Ranjan A. K. Kawase Y. Cheng R. K. Kara R. J. Bhattacharya R.. (2014). Cyclin a2 induces cardiac regeneration after myocardial infarction through cytokinesis of adult cardiomyocytes. Sci. Transl. Med. 6:224ra227. 10.1126/scitranslmed.3007668 24553388

[B68] Shiba Y. Gomibuchi T. Seto T. Wada Y. Ichimura H. Tanaka Y.. (2016). Allogeneic transplantation of ips cell-derived cardiomyocytes regenerates primate hearts. Nature 538, 388–391. 10.1038/nature19815 27723741

[B69] Singelyn J. M. Sundaramurthy P. Johnson T. D. Schup-Magoffin P. J. Hu D. P. Faulk D. M.. (2012). Catheter-deliverable hydrogel derived from decellularized ventricular extracellular matrix increases endogenous cardiomyocytes and preserves cardiac function post-myocardial infarction. J. Am. Coll. Cardiol. 59, 751–763. 10.1016/j.jacc.2011.10.888 22340268 PMC3285410

[B70] Snider P. Hinton R. B. Moreno-Rodriguez R. A. Wang J. Rogers R. Lindsley A.. (2008). Periostin is required for maturation and extracellular matrix stabilization of noncardiomyocyte lineages of the heart. Circ. Res. 102, 752–760. 10.1161/CIRCRESAHA.107.159517 18296617 PMC2754697

[B71] Swinehart I. T. Badylak S. F. (2016). Extracellular matrix bioscaffolds in tissue remodeling and morphogenesis. Dev. Dyn. 245, 351–360. 10.1002/dvdy.24379 26699796 PMC4755921

[B72] Traverse J. H. Henry T. D. Dib N. Patel A. N. Pepine C. Schaer G. L.. (2019). First-in-man study of a cardiac extracellular matrix hydrogel in early and late myocardial infarction patients. JACC Basic Transl. Sci. 4, 659–669. 10.1016/j.jacbts.2019.07.012 31709316 PMC6834965

[B73] Uriel N. Sayer G. Annamalai S. Kapur N. K. Burkhoff D. (2018). Mechanical unloading in heart failure. J. Am. Coll. Cardiol. 72, 569–580. 10.1016/j.jacc.2018.05.038 30056830

[B74] Vite A. Zhang C. Yi R. Emms S. Radice G. L. (2018). A-catenin-dependent cytoskeletal tension controls yap activity in the heart. Development 145:dev149823 10.1242/dev.149823 29467248 PMC5868989

[B75] Walsh S. Pontén A. Fleischmann B. K. Jovinge S. (2010). Cardiomyocyte cell cycle control and growth estimation *in vivo*–an analysis based on cardiomyocyte nuclei. Cardiovasc. Res. 86, 365–373. 10.1093/cvr/cvq005 20071355

[B76] Wang J. Karra R. Dickson A. L. Poss K. D. (2013). Fibronectin is deposited by injury-activated epicardial cells and is necessary for zebrafish heart regeneration. Dev. Biol. 382, 427–435. 10.1016/j.ydbio.2013.08.012 23988577 PMC3852765

[B77] Wang W. E. Li L. Xia X. Fu W. Liao Q. Lan C.. (2017). Dedifferentiation, proliferation, and redifferentiation of adult mammalian cardiomyocytes after ischemic injury. Circulation 136, 834–848. 10.1161/CIRCULATIONAHA.116.024307 28642276 PMC5575972

[B78] Wang X. Senapati S. Akinbote A. Gnanasambandam B. Park P. S. Senyo S. E. (2020). Microenvironment stiffness requires decellularized cardiac extracellular matrix to promote heart regeneration in the neonatal mouse heart. Acta Biomater. 113, 380–392. 10.1016/j.actbio.2020.06.032 32590172 PMC7428869

[B79] Wei K. Serpooshan V. Hurtado C. Diez-Cuñado M. Zhao M. Maruyama S.. (2015). Epicardial fstl1 reconstitution regenerates the adult mammalian heart. Nature 525, 479–485. 10.1038/nature15372 26375005 PMC4762253

[B80] Williams C. Quinn K. P. Georgakoudi I. Black L. D. III (2014). Young developmental age cardiac extracellular matrix promotes the expansion of neonatal cardiomyocytes *in vitro*. Acta Biomater. 10, 194–204. 10.1016/j.actbio.2013.08.037 24012606 PMC3840040

[B81] Wright K. M. Lyon K. A. Leung H. Leahy D. J. Ma L. Ginty D. D. (2012). Dystroglycan organizes axon guidance cue localization and axonal pathfinding. Neuron 76, 931–944. 10.1016/j.neuron.2012.10.009 23217742 PMC3526105

[B82] Wu C. C. Jeratsch S. Graumann J. Stainier D. (2020). Modulation of mammalian cardiomyocyte cytokinesis by the extracellular matrix. Circ. Res. 127, 896–907. 10.1161/CIRCRESAHA.119.316303 32564729

[B83] Xiang F. L. Guo M. Yutzey K. E. (2016). Overexpression of tbx20 in adult cardiomyocytes promotes proliferation and improves cardiac function after myocardial infarction. Circulation 133, 1081–1092. 10.1161/CIRCULATIONAHA.115.019357 26841808 PMC4792775

[B84] Yahalom-Ronen Y. Rajchman D. Sarig R. Geiger B. Tzahor E. (2015). Reduced matrix rigidity promotes neonatal cardiomyocyte dedifferentiation, proliferation and clonal expansion. Elife 4:e07455. 10.7554/eLife.07455.020 26267307 PMC4558647

[B85] Yap L. Tay H. G. Nguyen M. T. X. Tjin M. S. Tryggvason K. (2019). Laminins in cellular differentiation. Trends Cell Biol. 29, 987–1000. 10.1016/j.tcb.2019.10.001 31703844

[B86] Zebrowski D. C. Vergarajauregui S. Wu C. C. Piatkowski T. Becker R. Leone M.. (2015). Developmental alterations in centrosome integrity contribute to the post-mitotic state of mammalian cardiomyocytes. Elife 4:e05563. 10.7554/eLife.05563.014 26247711 PMC4541494

